# Sphingosine-1-phosphate attenuates proteoglycan aggrecan expression via production of prostaglandin E_2 _from human articular chondrocytes

**DOI:** 10.1186/1471-2474-8-29

**Published:** 2007-03-20

**Authors:** Kayo Masuko, Minako Murata, Hiroshi Nakamura, Kazuo Yudoh, Kusuki Nishioka, Tomohiro Kato

**Affiliations:** 1 Department of Bioregulation and Proteomics, Institute of Medical Science, St. Marianna University School of Medicine, Kanagawa, Japan; 2 Department of Frontier Medicine, Institute of Medical Science, St. Marianna University School of Medicine, Kanagawa, Japan

## Abstract

**Background:**

Sphingosine-1-phosphate (S1P), a downstream metabolite of ceramide, induces various bioactivities via two distinct pathways: as an intracellular second messenger or through receptor activation. The receptor for S1P (S1PR) is the family of Endothelial differentiation, sphingolipid G-protein-coupled receptor (EDG). We have here attempted to reveal the expression of EDG/S1PR in human articular chondrocytes (HAC), exploring the implications of S1P in cartilage degradation.

**Methods:**

Articular cartilage specimens were obtained from patients with rheumatoid arthritis (RA), osteoarthritis (OA) or traumatic fracture (representing normal chondrocytes) who underwent joint surgery. Isolated HAC were cultured *in vitro *by monolayer and stimulated with S1P in the presence or absence of inhibitors of signaling molecules. Stimulated cells and culture supernatants were collected and subjected to analyses using reverse transcription-polymerase chain reaction (RT-PCR), Western blotting, and enzyme-linked immunosorbent assay (ELISA).

**Results:**

All of the tested HAC samples showed positive results in terms of EDG/S1PR expression in basal condition. When HAC was stimulated with S1P, a significant increase in prostaglandin (PG) E_2 _production was observed together with enhanced expression of cyclooxygenase (COX)-2. S1P stimulated extracellular signal-regulated kinase (ERK) and p38 mitogen-activated protein kinase (MAPK) in HAC, and the PGE_2 _induction was abrogated by PD98059 and SB203580. *Pertussis toxin *inhibited the PGE_2 _induction from HAC by S1P, suggesting an essential role for Gi protein. S1P also attenuated the expression of proteoglycan aggrecan, a component of cartilage matrix, in HAC at transcriptional level.

**Conclusion:**

It was suggested that the S1P-induced PGE_2 _was at least in part involved in the aggrecan-suppressing effect of S1P, seeing as COX inhibitors attenuated the effect. Accordingly, S1P might play an important role in cartilage degradation in arthritides.

## Background

Sphingosine-1-phosphate (S1P) is a bioactive sphingolipid metabolite formed by phosphorylation of sphingosine through activation of sphingosine kinase (reviewed in [1]). S1P exhibits pleiotropic functions, such as cell growth, cell differentiation, survival, angiogenesis, cell migration, and the regulation of immune functions [1,2].

Although S1P is released mainly from platelets, other cell types, such as erythrocytes or mononuclear cells, can also produce S1P [3] and high concentrations of S1P can be found in human sera (i.e. in nanomolar to micromolar concentrations) [1,4]. S1P functions via two distinct pathways: as an intracellular second messenger or through activation of specific G protein-coupled receptors. The receptors for S1P are referred to as S1PR_s_, and these include the family of endothelial differentiation, lysophosphatidic acid G-protein-coupled receptors (EDG) so far identified [1], i.e. EDG1/S1P_1_, EDG5/S1P_2_, EDG3/S1P_3_, EDG6/S1P_4_, and EDG8/S1P_5_. Functional redundancy among the EDG receptors has been reported. In fact, it has been reported that different cells express different EDG receptors, and S1P can potentially stimulate diverse signals in a variety of cell types as well as within the same cell. This raises the possibility of diverse biological outcomes [2]. For example, although S1P in general has mitogenic potential, it may also have antiproliferative potential in certain cell types[5,6].

Osteoarthritis (OA) is a degenerative joint disease in which the aging process and repeated mechanical load on the joint are thought to play a key role. Recent investigations, however, have shed light on the inflammatory aspects of OA pathogenesis, involving various arrays of inflammatory mediators such as prostaglandins (PG) [7]. For example, PGE_2_, a representative PG, has been suggested as a possibly catabolic factor in cartilage. In this context, we and others have identified expressions of PGE_2 _synthases in OA chondrocytes [8,9], suggesting a PG-mediated degenerative process for cartilage in OA.

Although S1P has been reported to induce production of PGE_2 _in several cell types via the activation of cyclooxigenase (COX)-2 [5,10-12], its role in human chondrocytes is still not known. Here we have attempted to clarify the possible role of S1P in cartilage in HAC, focusing on its potential to induce PGE_2 _in chondrocytes.

## Methods

### Cells

HAC were obtained from patients with osteoarthritis (OA; N = 41, M/F = 8/33, age 55–86 [mean 77.7]), rheumatoid arthritis (RA; N = 14, M/F = 1/13, age 45–76 [mean 56.8]), or traumatic fracture (N; N = 11, M/F = 0/11, age 69–92 [mean 79.8]) who underwent arthroplasty of a knee or hip joint. The diagnosis of OA was made according to the criteria of Kellgren and Lawrence [13]. RA was classified according to the criteria of the American College of Rheumatology [14]. Cartilage samples obtained from patients with traumatic fracture were largely normal and no significant pathological changes were observed. These samples were therefore designated as "normal". Written informed consent was obtained from each patient and the study protocol was approved by our institution's ethical committee. The study was performed in compliance with the World Medical Association Declaration of Helsinki (1964).

After careful removal of synovial tissue, cartilage was minced, washed and treated with collagenase. Isolated chondrocytes were then washed and cultured *in vitro *by monolayer in DMEM medium supplemented with 10 % fetal calf serum (FCS) and antibiotics. The attached cells (P0) were expanded on type I collagen-coated culture dishes and the cells at subconfluent (P1 cells) were used in the experiments. The differentiated phenotypes of the cells used in the experiments were confirmed through macroscopic observation and by expressions of type II collagen and aggrecan mRNA (data not shown).

### Reagents

Sphingosine-1-phosphate (S1P) was purchased from Cayman Chemical Co. (Ann Arbor, M, USA). Recombinant human IL-1 was purchased from R&D Systems, Inc. (Minneapolis, MN, USA). All the following inhibitors were obtained from Calbiochem by Merck Ltd. (Tokyo, Japan): SB203580, PD98059, SP600125, and LY294002.

Anti-EDG5 antibody of mouse monoclonal IgG was purchased from Oncogene Research Products (San Diego, CA, USA), and used for western blot. For immunohistochemistry, anti-EDG5, rabbit-polyclonal antibody (Genesis Biotech Inc., Taiwan, R.O.C.) was used.

Anti-COX-2 antibody was purchased from Cayman Chemical Company. Anti-p38 mitogen-activated protein kinase (MAPK), anti-phosphorylated p38 MAPK, anti-ERK, anti-phosphorylated extracellular signal-regulated kinase (ERK)-1/2, anti-c-Jun N-terminal kinase (JNK1, JNK2), and anti-phosphorylated JNK and anti-β-actin antibodies were purchased from Sigma (Saint Louis, MO, USA). Anti-glyceraldehyde 3-phosphate dehydrogenase (GAPDH) was purchased from Abcam Ltd. (Cambridge, UK).

### In vitro stimulation of chondrocytes

Chondrocytes were serum-starved in a medium with 0.5 % FCS for 24 h prior to the experiments and either stimulated or not with IL-1 (10 ng/ml) or S1P for indicated periods. Where specified, cells were pretreated with pertussis toxin from *Bordetella pertussis *(Sigma) for 18 h, and SB203580 (10 μM; p38 MAPK inhibitor), PD98059 (50 μM; ERK1/2 inhibitor), SP600125 (10 μM; JNK inhibitor II), or LY294002 (50 μM; phosphatidylinositol 3-kinase (PI3K) inhibitor), Meloxicam (10 μM) or Indomethacin (25 μM) for 1 h before the addition of S1P. Cell viability was not affected by S1P (up to 50 μM), the vehicle, or any of the inhibitors during the culture period, as confirmed by trypan blue exclusion, flow cytometry or a cell viability assay (CellTiter 96™ Aqueous Non-Radioactive Cell Proliferation Assay; Promega CO., WI, USA)(data not shown).

Stimulated chondrocytes and culture supernatants were collected and subjected to the following analyses.

### Preparation of specimens of cartilage, and immunohistochemistry

Articular cartilage sections were prepared as previously described [15]. Briefly, cartilage slices were fixed in 4 % paraformaldehyde/0.01 M phosphate-buffered saline. After decalcification, the sections were embedded in paraffin and 5 μm sections were prepared.

After deparaffinization, sections were incubated with proteinase K (DAKO, USA) and Block Ace™ (Dainippon Pharmaceuticals, Osaka, Japan), followed by incubation with either anti-EDG5 antibody (Genesis Biotech Inc., Taiwan, R.O.C.) or rabbit immunoglobulin fraction (DakoCytomation, Glostrup, Denmark) for 1.5 h at room temperature. Samples were then processed and visualized with DAKO LSAB+kit/HRP (DAKO) in line with the manufacturer's instructions.

### RT-PCR

Total RNA was isolated from the cultured cells using RNAbee™ (Tel-Test, Inc., Friendswood, TX, USA) in accordance with the manufacturer's instructions. Expression of mRNA of the EDG receptors was analyzed by reverse transcription-polymerase chain reaction (RT-PCR) using the specific primer sets (Table 1). The PCR conditions were as follows: initial denaturation at 94°C, 2 min; 35 cycles of denaturation at 94°C, 1 min; amplification at 58°C, 1 min; extension at 72°C, 1 min; and final extension at 72°C, 5 min.

The size of amplified products was checked by agarose gel electrophoresis. For semi-quantitive analyses, relative band intensities against internal control were calculated using ImageGauge software (FujiFilm, Tokyo, Japan).

### Real-time PCR

Quantitative analysis of mRNA expression for aggrecan gene was performed using the SYBR Premix Ex Taq™ system and LightCycler (Roche Diagnostic) according to the manufacturer's instructions. The sequences of the primers used for the Real-time PCR were as follows:

GAPDH: 5') GCACCGTCAAGGCTGAGAAC

3') ATGGTGGTGAAGACGCCAGT

aggrecan: 5') ACCCTGGAAGTCGTGGTGAAAG

3') CGTGGCAATGATGGCACTG

### ELISA

Level of PGE_2 _in the supernatants was quantified using an EIA kit (Cayman Chemical Co., Ann Arbor, MI, USA). Aggrecan release into the supernatants was measured using a Human Aggrecan (Proteoglycan) EASIA™ ELISA Kit (BioSource International, Inc.; Camarillo, CA, USA).

### Western blotting

Whole cell lysates were extracted from the cultured cells using standard lysis buffer (20 mM Tris-HCl, 250 mM NaCl, 1% NP-40, 1 mM dithiothreitol, 10 mM NaF, 2 mM Na_3_VO_4_, and 10 mM Na_4_P_2_O_7_) and stored at -30°C prior to use. Protein concentration was determined using the Bradford method (Bio-Rad protein assay reagent; BioRad Laboratories, Hercules, CA). The lysates were mixed with migration dye and applied to the SDS-polyacrylamide gel electrophoresis (PAGE) gel. After transfer to a polyvinylidine difluoride membrane, a primary antibody (as noted in ''Reagents'') was added. The membrane was then washed and it reacted with the corresponding second antibody. Finally, the signals were visualized with the ECL system (Amersham Biosciences). Densitometry of the signal bands was analyzed using ImageJ software [16].

Statistical significance in each experiment was analyzed using Prism4™ for Macintosh software (GraphPad Software Inc., San Diego, CA, USA)

## Results and discussion

### Human articular chondrocytes express EDG/S1P receptors

**Figure 1:**
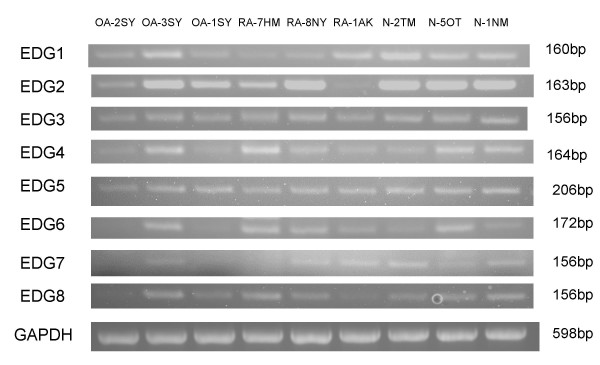
RT-PCR analyses for the EDG family receptors of S1P in HAC. Chondrocyte samples were analyzed for the expression of EDG/S1P receptor families. OA: osteoarthritis; RA: rheumatoid arthritis; N: non-arthritic traumatic fracture; numbers and alphabets identify each patient.

**Figure 2:**
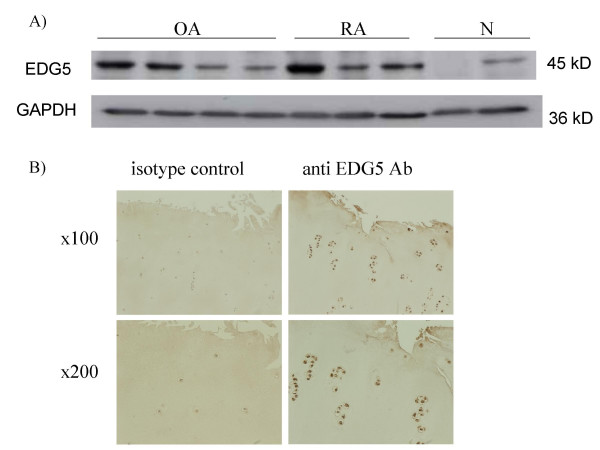
EDG5 expression in HAC. A) Western blot: Whole cell lysates from cultured HAC were subjected to immunoblotting. EDG5 was detected as a distinct band at 45 kD. B) Immunohistochemistry. Specimens of OA cartilage were subjected to analysis of EDG5 expression at protein level. The representative images are shown (N = 3).

This is, to our knowledge, the first study to show the expression of EDG/S1P receptors in HAC. As shown in the representative results of RT-PCR in Figure 1, regardless of the origin of cartilage (from RA, OA or normal joints), essentially all of the tested HAC samples expressed most of the tested EDG families at basal level. We also confirmed the expression of representative receptor, EDG5, at the protein level (Fig. 2A), and also by immunohistochemistry using articular cartilage samples (Fig. 2B).

As indicated, each individual showed a different pattern of EDG/S1PR family expression. Bearing in mind ligand-receptor redundancy, variations in receptor expression in HAC may lead to distinct patterns of response to variable stimuli for each individual.

### S1P increases PGE_2 _production from chondrocytes via EDG receptor

To explore the effect of S1P on human chondrocytes, we cultured HAC with S1P (at 0.1 to 10 μM) *in vitro*. The treatment did not induce proliferation, or negatively impact the viability of the cultured HAC, for up to 48 h (data not shown). This seems to be in contrast to findings in rat chondrocytes, where S1P induced cellular proliferation via ERK MAPK[17], possibly attributable to differences in response to S1P between the species.

**Figure 3:**
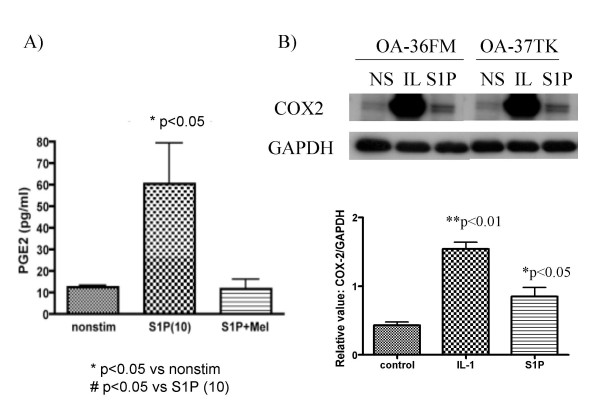
S1P stimulation induces PGE_2 _production from HAC. A): OA chondrocytes were stimulated *in vitro *with S1P (10 μM) with or without meloxicam (Mel; 1 μM) for 24 h, and levels of PGE_2 _in the culture supernatants were subjected to ELISA in triplicate (N = 3 each). B) Expressions of COX-2 were analyzed with western blot (representative results are shown). The intensities of bands detected with COX-2 analysis were calculated according to their relative ratio against GAPDH (N = 5).

**Figure 4:**
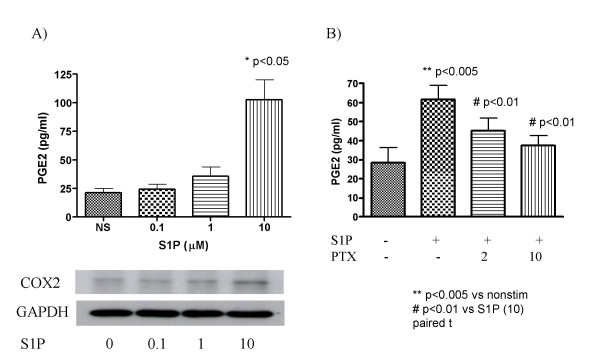
Dose-response and involvement of GPCR in the PGE_2 _production by S1P. A) Dose-response: OA chondrocytes were stimulated *in vitro *with S1P (0.1, 1 or 10 μM) for 24 h. Top: Levels of PGE_2 _in the culture supernatants were assayed using ELISA in triplicate (N = 3 each). Bottom: A representative result of western blot of COX-2 expression is shown. B): PTX inhibition: OA chondrocytes were stimulated *in vitro *with S1P (10 μM) with or without pertussis toxin (PTX, 10 ng/ml) for 24 h, and levels of PGE_2 _were analyzed using ELISA (N = 3 each, in triplicate).

Lipid mediators such as ceramide, sphingosine or S1P have been demonstrated to induce PGE_2 _in several cell types [5,10-12], a possibly catabolic mediator in articular cartilage [8,9,18]. We here found that S1P significantly increased prostaglandin E_2 _(PGE_2_) production from HAC (Fig. 3A). The level of PGE_2 _(basal or induced) showed considerable variance between samples, but there was a consistent tendency toward increased PGE_2 _production after S1P stimulation (Fig. 3A and data not shown). We also observed that HAC cultured in medium containing fatty acid-free BSA instead of 0.5 % FCS showed a similar increase in PGE_2 _after S1P stimulation (data not shown). The S1P-induced PGE_2 _production was almost completely abrogated by Meloxicam, a preferential inhibitor of COX-2 (Fig. 3A); and the induction of COX-2 expression by S1P was confirmed by Western blot (Fig. 3B). The enhancing effect of S1P on PGE_2 _and COX-2 induction was observed as dose-dependent between concentrations at 0.1 to 10 μM (Fig. 4A), and was increased as time-dependent to reach the maximum level at 24 h after stimulation (data not shown).

Another assay showed that the addition of a Gi protein inhibitor, *pertussis toxin *(PTX), suppressed the induction of PGE_2 _by S1P (Fig. 4B). Thus it was suggested that PGE_2 _induction by S1P depends on COX-2 and signals via G-protein-coupled receptors (GPCR), i.e. EDG/S1PR, respectively; of particular interest was the role of Gi protein in the S1P-induced PGE_2 _production in HAC.

### The PGE_2 _induction by S1P is mediated via p28 and ERK MAPKs

**Figure 5:**
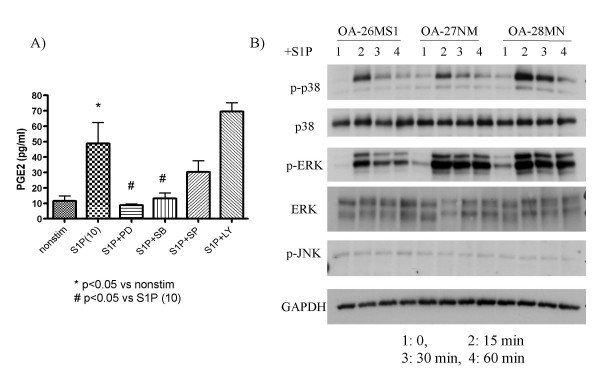
MAPK-mediated signaling in the S1P-induced PGE2 production by HAC. A) Inhibition assays: OA chondrocytes were stimulated *in vitro *with S1P (10 μM) for 24 h with or without pre-treatment with inhibitor, and levels of PGE_2 _in the culture supernatants were subjected to ELISA in triplicate (N = 5). SB: SB203580 (10 μM; p38 MAPK inhibitor), PD: PD98059 (50 μM; ERK1/2 inhibitor), SP: SP600125 (10 μM; JNK inhibitor II), LY: LY294002 (50 μM; PI3K inhibitor). B) Western blot: Chondrocytes (N = 3) were stimulated *in vitro *with S1P (10 μM) for the indicated time period, and the lysates were analyzed for activations of signaling molecules.

As Kim et al. recently reported on the key role of p38 and ERK MAPKs in S1P-induced proliferation in rat chondrocytes[17], we here explored the intracellular signaling mechanism of S1P-induced PGE_2 _production in human chondrocytes. Results (Fig. 5A, B) of the inhibition assays demonstrated that the S1P-induced PGE_2 _production in HAC was abrogated by PD98059 and SB203580, indicating the dominant role of ERK1/2 and p38 MAPK; the JNK inhibitor SP600125 and the PI3K inhibitor LY294002 did not alter the level of PGE_2 _production significantly (Fig. 5A). S1P-induced activation of p38 and ERK MAPK, but not of JNK, was also detected by western blotting (Fig. 5B). These findings indicate that p38 and ERK MAPK are dominantly responding kinases following S1P stimulation in both rat and human cells leading to downstream phenomenon such as proliferation and prostanoid production.

### S1P attenuates proteoglycan aggrecan synthesis by HAC

An important function of articular chondrocytes is to synthesize components of the extracellular matrix of cartilage, including proteoglycan and type II collagen. To investigate whether S1P would affect the matrix synthesis by HAC, we measured secreted aggrecan as well as expression of aggrecan mRNA in HAC stimulated with S1P.

**Figure 6:**
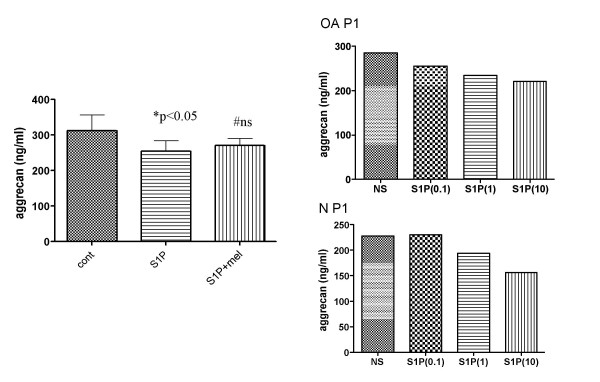
S1P attenuates aggrecan synthesis in HAC. Aggrecan ELISA. Left: OA chondrocytes were stimulated *in vitro *with S1P (10 μM) for 24 h and the supernatants were analyzed for aggrecan secretion. Right: OA chondrocytes (top) and a nonarthritic chondrocytes (bottom) were stimulated with S1P at the indicated concentrations.

**Figure 7:**
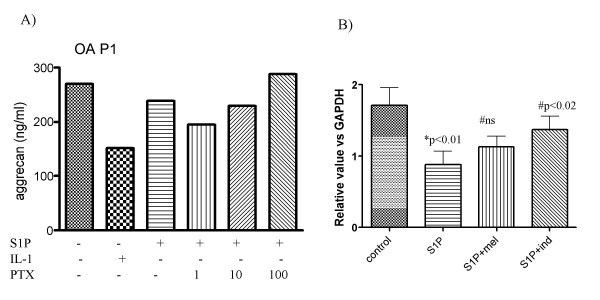
Effect of PTX and COX inhibitors in the aggrecan suppression by S1P. A) PTX partly abrogate the suppressing effect of S1P. The result of an assay on an OA sample is shown. B) Real-time PCR: aggrecan mRNA. OA chondrocytes were stimulated *in vitro *with S1P (10 μM) for 24 h and the mRNA was analyzed for aggrecan expression (N = 3). mel: meloxicam (1 μM), ind: indomethacin (25 μM).

The results showed that aggrecan secretion was attenuated in HAC stimulated with S1P (Fig. 6, left). We analyzed the dose-dependency of the suppressive effect with S1P using an OA sample and a non-arthritic sample, and both samples showed a tendency toward dose-dependence (Fig. 6, right). In the assay using an OA sample, pre-treatment with PTX helped the level of aggrecan secretion partly recover (Fig. 7A). In this regard, as it was found that the chondrocyte samples tested showed differing patterns of EDG/S1PR expression (see Fig. 1), the specificity of the EDG/S1PR family in the aggrecan-suppressing effect might be sample- or patient-dependent. Further assays using a sufficient number of samples, as well as inhibitors for each EDG/S1PR receptor, will therefore be needed to understand the mechanism in more detail.

Since we were unable to detect any significant upregulation of mRNA among representative aggrecanases (i.e. ADAMTS (a disintegrin and metalloproteinase domain with thrombospondin motif) -1, 4, and 5; data not shown), we hypothesized that the suppression of aggrecan expression would, at least in part, be due to an inhibition of aggrecan transcription. The results of quantitative PCR (Fig. 7B) supported this hypothesis; S1P significantly suppressed the expression of aggrecan mRNA in HAC. Meloxicam slightly abrogated the suppressing effect of S1P, while Indomethacin promoted more effective recovery of the aggrecan expression than Meloxicam (Fig. 7B).

As for the implication of lipids in proteoglycan degradation in cartilage, Sabatini *et al *reported that ceramide, an upstream lipid mediator of S1P, might contribute to chondrocyte apoptosis [19] and also to cartilage degradation through the induction of aggrecanase activity[20]. Thus, the possible impact of sphingolipids on the aggrecanases could have importance beyond the regulation of gene expression. Since the expression of sphingosine kinase (SPHK), an enzyme essential for converting sphingosine to S1P, was reported to be enhanced in both OA and RA synovial tissues [21], sphingolipids might also play an important role in the pathogenesis of OA [17] and RA[22].

Although a possible suppressing role of COX-2/PGE_2 _in aggrecan expression has been suggested, the precise mechanism by which the prostanoids might suppress aggrecan transcription is still unclear. In our results, the nonselective COX inhibitor indomethacin (blocks COX-1 and COX-2) was slightly more effective than meloxicam (a COX-2-preferential inhibitor) (Fig. 7B), implying that the amount of COX-induced PGE_2_, and/or a balance between COX-1 (constitutive) and COX-2 (inducible) activations might be factors involved in the S1P-mediated signaling in HAC. Although the direct effect of PGE_2 _in proteoglycan degradation might still cause controversy [23], there would appear to be a synergy between IL-1, S1P and PGE_2_.

In conclusion, we have here reported, for the first time, the possible involvement of the S1P in cartilage degradation. Since S1P has been detected in sera, the findings of *in vitro *experiments ought to be at least partly representative of the physiological bioactivity of S1P in inflammatory or degenerative articular joints *in vivo*. In fact, a recent report described how S1P could be detected in synovial fluid of RA and OA in higher concentrations than in sera [22]. A possible relationship between chondrocytes and sphingolipids would provide a novel tool to help us analyze and possibly reverse the degradative pathway in cartilage structure in arthropathy.

## Conclusion

Human articular chondrocytes express the EDG/S1PR receptors for S1P, and respond to S1P stimulation by inducing a significant increase of PGE_2_via COX-2 and MAPK. It has been suggested that S1P-induced PGE_2 _is involved in the aggrecan-suppressing effect of S1P. The sphingolipid mediator S1P may thus play an important role in cartilage degradation in arthritides such as osteoarthritis.

## Competing interests

The author(s) declare that they have no competing interests.

## Authors' contributions

KM designed and carried out the studies. MM and HN participated in the Immunohistochemistry. KY participated in the immunoassays. KN participated in coordination of the study. TK participated in the design. All authors read and approved the final manuscript.

## Pre-publication history

The pre-publication history for this paper can be accessed here:



## Figures and Tables

**Table 1:** primer sequences

specificity	sense (5'-3) antisense (5'-3)	bp	Ref. [GenBank accession nos]
EDG1	TCC TTC ACT TTA GTT TCA AAC CCA AGT	160	[GenBank:NM-001400]
	CAA CCC CAG CTC TGA TAA CTC TCA		
EDG2	ATT TTG TGT GGT TTG GTG CAA GTC	163	[GenBank:NM-057159]
	AGC AGT CCA TAG TCT CAT CCA TCA C		
EDG3	AAA ATT AGC TGG ATG CGA TAG CAC	156	[GenBank:NM-005226]
	TTT GAG ACA GAG GGT CGC TCT GT		
EDG4	CTG ACT GGC AGG ACA AGG TCT G	164	[GenBank:NM-004720]
	ACC CCC TAA ACC TGA GAT GGC T		
EDG5	CCA AGC ATT ATG TGC TGT GC	206	[GenBank:NM-004230]
	CAG AAG GAG GAT GCT GAA GG		
EDG6	CTA CTC CAA GCG CTA CAT CCT CT	172	[GenBank:NM-003775]
	GAT CAT CAG CAC CGT CTT CAG		
EDG7	CTC ATC ATG GTT GTG GTG TAC CTG	156	[GenBank:NM-012152]
	GCA TAC CAC AAA CGC CCC TAA G		
EDG8	AGG ACC TTG TGG GTG ATA TAG AGG AC	156	[GenBank:NM-030760]
	CCC CTT CAC CTT CTC TGG TTT TC		
COX2	TTC TGA GAT TGT GGG AAA ATT GCT	305	[GenBank:M90100]
	AGA TCA TCT CTG GCT GAG TAT CTT		
GAPDH	CCA CCC ATG GCA AAT TCC ATG GCA	598	[GenBank:NM-002046]
	TCT AGA CGG CAG GTC AGG TCC ACC		[24]
